# Effects of 7.5% carbon dioxide (CO_2_) inhalation and ethnicity on face memory

**DOI:** 10.1016/j.physbeh.2015.04.027

**Published:** 2015-08-01

**Authors:** Angela S. Attwood, Jon C. Catling, Alex S.F. Kwong, Marcus R. Munafò

**Affiliations:** a MRC Integrative Epidemiology Unit (IEU) at the University of Bristol, United Kingdom; b UK Centre for Tobacco and Alcohol Studies, University of Bristol, United Kingdom; c School of Experimental Psychology, University of Bristol, United Kingdom; d School of Psychology, University of Birmingham, United Kingdom

**Keywords:** Anxiety, 7.5% CO_2_, Eyewitness memory, Face recognition, Other-ethnicity effect

## Abstract

The ability to accurately verify facial identity has important forensic implications, but this ability is fallible. Research suggests that anxiety at the time of encoding can impair subsequent recall, but no studies have investigated the effects of anxiety at the time of recall in an experimental paradigm. This study addresses this gap using the carbon dioxide (CO_2_) model of anxiety induction. Thirty participants completed two inhalations: one of 7.5% CO_2_-enriched air and one of medical air (i.e., placebo). Prior to each inhalation, participants were presented with 16 facial images (50% own-ethnicity, 50% other-ethnicity). During the inhalation they were required to identify which faces had been seen before from a set of 32 images (16 seen-before and 16 novel images). Identification accuracy was lower during CO_2_ inhalation compared to air (*F*[1,29] = 5.5, *p* = .026, *η*_*p*_^2^ = .16), and false alarm rate was higher for other-ethnicity faces compared to own-ethnicity faces (*F*[1,29] = 11.3, *p* = .002, *η*_*p*_^2^ = .28). There was no evidence of gas by ethnicity interactions for accuracy or false alarms (*p*s > .34). Ratings of decision confidence did not differ by gas condition, suggesting that participants were unaware of differences in performance. These findings suggest that anxiety, at the point of recognition, impairs facial identification accuracy. This has substantial implications for eyewitness memory situations, and suggests that efforts should be made to attenuate the anxiety in these situations in order to improve the validity of identification.

## Highlights

• Use of carbon dioxide challenge to investigate acute anxiety effects on face memory
• Investigation of the “own-ethnicity” effect and its interaction with acute anxiety
• Results show decreased accuracy for face memory during acutely anxious states.
• Results show increased false identifications when viewing other ethnicity faces.
• Efforts should be made to attenuate anxiety in eye witness situations.

## 1 Introduction

The ability to verify facial identity plays a vital role in a number of security and forensic settings. However, both anecdotal and empirical reports indicate that this ability is highly fallible [1,2]. Unsurprisingly, a large body of research has investigated factors which affect the reliability of face recognition [3–5] and eyewitness memory [6–10]. One key factor that has received substantial empirical attention is the role of stress. While there are data showing the effects of stress at the time of encoding, relatively little experimental research has investigated how stress responses at the point of *recall* affect identification performance.

There is evidence from face recognition studies that pharmacological challenges such as oxytocin administration alter face recognition ability [11], but relatively few studies have investigated the effects of acute anxiety on this ability. Using a face recognition paradigm, Li and colleagues [3] reported differences in neural response to emotional faces following a psychosocial stress task, but no change in recognition performance. In contrast, more research has examined the effects of stress on eyewitness memory, but findings have been mixed [12–16]. In a meta-analytical review, Deffenbacher, Bornstein, Penrod and McGorty [17] concluded that high levels of stress at the time of encoding negatively affect both accuracy in subsequent identification and the recall of crime-related details. While the data indicated that performance was degraded in both target present and target absent line-ups, effect sizes were larger when the target was present. The authors suggested that previous inconsistency in findings may be due to individuals not experiencing the full range of anxiogenic responses to the stressful event. It is possible that the aforementioned positive effects reflect increases in arousal and attentional orienting that are free of the negative subjective, cognitive and physiological aspects of anxiety. This was supported by the observation that average heart rates in response to the stressor were comparable to normal resting rates.

Therefore, to fully understand how performance alters as a function of stress in the real world, laboratory models need to simulate the full anxiogenic response that is associated with a stressful episode; in particular subjective feelings of anxiety. However, inducing comprehensive and reliable increases in anxiety under laboratory conditions has been a challenge. Historically, many studies have used psychosocial stressors such as the Trier Social Stress Test [18], which requires participants to talk in front of a panel of “experts”. While these tasks have utility, they also have a number of important limitations. First, aspects of the stress response (particularly the physiological parameters) are subject to individual variation. Second, it is practically difficult to deliver cognitive tasks during peak anxiety (i.e., when the individual is giving a presentation).

To circumvent some of these issues, respiratory challenges have been used which involve inhalation of air that has heightened levels of carbon dioxide (CO_2_). This is a physiological challenge that reliably induces both physiological and subjective symptoms of anxiety. Importantly, it has also been shown to induce cognitive and attentional changes that are characteristic of general anxiety including enhanced threat processing [19,20] and attentional orienting function [21]. Furthermore, inhalation of 7.5% CO_2_ can be sustained for up to 20 min, which enables tasks to be completed during the period of peak response. To date only one study has used this model to directly examine the effects of acute anxiety on facial identification [22]. In this study, a simultaneous face-matching paradigm was used in which participants were required to identify whether two photographs showed the same unfamiliar face. Compared to air (i.e., placebo), 7.5% CO_2_-inhalation was associated with reduced accuracy in same-face identification (hits). However, there was no difference between air and CO_2_ inhalation on false alarm rate (i.e., incorrectly identifying that two different faces were the same individual).

In this study, we have extended this work by investigating the effects of acute anxiety induced by 7.5% CO_2_ on memory of unfamiliar faces. This is an important line of investigation as previous research has focussed on the effects of anxiety at the time of encoding but little is known about how anxiety may affect performance at the point of recognition. This has important implications for eyewitness situations, as witnesses may experience acute anxiety during identification, particularly if they are faced with the perpetrator of a violent crime. A standard face recognition task was used in this study, which comprised two phases (encoding and recognition). The first phase involved individuals passively viewing a series of photographed faces prior to the inhalation. The second phase was completed during the inhalation and presented previously-seen images along with an equal number of previously-unseen images. Participants were asked to indicate whether they have seen each individual before. All participants completed two comparable versions of the task (one during inhalation of air and one during inhalation of 7.5% CO_2_-enriched air).

We also investigated the cross-ethnicity effect (i.e., recognition of own-ethnicity faces is superior to recognition of other-ethnicity faces), which has important implications for forensic situations and the justice system. The effect itself is well supported [23–25], and findings from the face recognition and eyewitness literature have consistently shown that viewers are more likely to misidentify someone of another ethnicity compared to their own ethnicity [23,26]. The effect, however, is not uniform or inevitable. It can be moderated by a number of factors including facial distinctiveness and experience with other ethnicities [26]. To date no research has directly investigated whether the effect is exacerbated by acute anxiety. Previous research has suggested that positive emotions eliminate the cross-ethnicity effect [27] and that the detrimental effects of anxiety on cognitive performance may be greater when there is greater task difficulty [28,29]. These findings indicate that negative affect induced by anxiety and the increased difficulty of other-ethnicity judgements may result in an enhanced other-ethnicity effect when anxious, but this needs to be tested empirically. Based on previous research, we hypothesised that acute anxiety induced by 7.5% CO_2_ inhalation would result in poorer face memory performance and that this effect would be greater for other-ethnicity faces.

## 2 Materials and methods

### 2.1 Design

This study was a repeated-measures design, with gas (air, 7.5% CO_2_) and face ethnicity (own-ethnicity, other-ethnicity) as within-subject factors. The order of gas inhalation and task version was counterbalanced across participants.

### 2.2 Participants

A total of 30 healthy volunteers (50% male) were recruited from the University of Bristol staff and students, and the local community. Participants were required to be aged between 18 and 40 years, of European ancestry, and in good physical and psychiatric health (assessed by a short structured interview based on the Mini-International Neuropsychiatric Interview; MINI). Additional exclusion criteria included recent use of prescribed medication (within 8 weeks of study session), daily smoking, pregnancy (verified by urine screen) or breast feeding, asthma, history of migraine and recent use of illicit drugs (verified by urine screen). Diastolic blood pressure (DBP: < 90 mm Hg) and systolic blood pressure (SBP: < 140 mm Hg), heart rate (50–90 bpm) and body mass index (BMI; 18–30 kg/m^2^) were required to be within the normal range. Prior to the study, participants were asked to refrain from alcohol for 36 h and (if a smoker) to refrain from smoking for 12 h. Participants were reimbursed £25 for their time at the end of testing. The study was conducted in line with the Declaration of Helsinki (2008), and was reviewed and approved by the University of Bristol Faculty of Science Research Ethics Committee (reference: 2702146564). All participants gave full written informed consent.

### 2.3 Measures and materials

Participants were tested for alcohol and carbon monoxide levels in exhaled breath using an AlcoDigital 3000 breathalyser (UK Breathalysers, UK) and a PiCo Smokerlyser (Bedfont Scientific Ltd., Maidstone, UK) respectively. Blood pressure and heart rate were recorded using an OMRON M6 Comfort Digital Blood Pressure Monitor (OMRON Healthcare Ltd., Milton Keynes, UK). Fresh urine samples were provided to test for the presence of barbiturates, benzodiazepines, opiates, tetrahydrocannabinol, methamphetamine, amphetamine and cocaine, and to test for pregnancy in female participants (SureScreen Diagnostics Ltd., Derby, UK).

#### 2.3.1 Questionnaires

Participants completed the Spielberger State-Trait Anxiety Inventory state (STAI-S) and trait (STAI-T) subscales, Anxiety Sensitivity Inventory (ASI) [30], Eysenck Personality Questionnaire Revised (EPQ-R) [31] and the Positive and Negative Affect Schedule [32].

#### 2.3.2 Task

As the task was completed twice by each participant (i.e., once during each inhalation), two comparable versions of the task were produced. The only difference between task versions was the facial images used. All images were taken from the Center for Vital Longevity Face Database [33]. They were of male European (own-ethnicity) or South Asian (other-ethnicity) individuals, aged between 18 and 46 years and displaying a neutral emotional expression.

The task comprised two phases: encoding and recognition. In the encoding phase, 16 (50% own-ethnicity, 50% other-ethnicity) images were displayed individually on screen. Each image was displayed for 2000 ms with inter-trial intervals of 500 ms. Participants were told to passively view the images and that there would be a memory test later in the session. The recognition phase of the task presented 32 images, 50% of which had been seen previously in phase one (balanced for ethnicity). The remaining 50% of images presented in phase two were novel facial images (balanced for ethnicity). On each trial, participants were required to identify whether they had seen the face previously using designated keyboard keys. Each image appeared on screen for 10,000 ms or until a response was made. After each trial, the image was replaced with a rating scale that asked participants to rate how confident they were in their decision (on 7-point scale ranging from “not confident” to “very confident”). The scale remained on screen until a response was made. There was an inter-trial interval of 1500 ms comprising 500 ms of a blank screen and 1000 ms of a screen displaying the word “Ready?”.

#### 2.3.3 Gas mixtures

The gas mixtures used were 7.5% CO_2_/21% O_2_/71.5% N and medical air (21%O_2_; BOC Ltd.). These were administered using an oro-nasal mask (Hans Rudolph, Kansas City, MO, USA), which was attached to a 500 L bag with tubing. For safety reasons, the gas was administered single-blind.

### 2.4 Procedure

Participants completed an initial telephone screening before attending the test session, in order to ascertain likely eligibility for the study. Eligible participants were scheduled to attend two test sessions, approximately one-week apart. Upon arrival at the first test session, participants gave written informed consent before completing a further screening procedure that included assessments of recent smoking (expired CO), recent alcohol consumption (breath test), pregnancy (urine screen), recent drug use (urine screen and self-report), neuropsychiatric health (MINI), height, weight, blood pressure and heart rate. If eligible, participants were then enrolled onto the study. A truncated version of the screening procedure (comprising physiological assessments listed above and self-report of any other relevant change) was conducted at the start of session two.

The study sessions (i.e., post-screening) lasted approximately 1.5 h each. One of the sessions involved inhalation of 7.5% CO_2_-enriched air and the other involved inhalation of medical air (placebo). The order of inhalations was counterbalanced across participants. At each session, baseline measures of heart rate and blood pressure were taken. Participants then completed phase one of the task, in which they were told to remember these faces for a later recognition test. Questionnaire measures of anxiety and mood (STAI-S, STAI-T, ASI, PANAS) were then completed. Twenty minutes after the end of phase one of the task, participants began the inhalation.

Once participants were fitted with the mask, the researcher read safety information aloud to the participants and advised them that they could stop the inhalation at any time. Participants were required to breathe the gas for 2 min prior to the start of the task to allow time for the anxiogenic response to initiate and stabilise. The second phase of the task was then completed and inhalations lasted for the duration of the task (approximately 4 min). Blood pressure and heart rate were recorded immediately after each inhalation. Participants completed state measures of anxiety and mood (STAI-S, PANAS) after each inhalation, but were told to rate on the basis of how they felt during the inhalation (when effects were at their strongest). After the inhalation, participants were required to stay in the laboratory with the researcher for a further 30 min to allow time for recovery. During this time, participants completed the EPQ-R (session two only). After 30 min, participants were asked to verbally report whether he/she was feeling back to normal and blood pressure and heart rate were checked to ensure they were in a normal range. Participants were then debriefed and reimbursed.

### 2.5 Data analysis

All analyses were performed using SPSS Statistics version 21 (SPSS Inc., Chicago IL, USA). The data that form the basis of the results presented here are archived on the University of Bristol Research Data Repository, http://dx.doi.org/10.5523/bris.1kxxqkrjxndf31ton5n64q8979. Sample size was determined from effect sizes obtained in previous research investigating the effects of CO_2_ inhalation on face matching ability [22]. This analysis was based on our primary outcome of interest (i.e., main effect of gas on face recognition). In a previous study there was an effect size of *d* = 0.53 for the reduction of face match accuracy on CO_2_ compared to air. Based on these data we required a sample size of 30 in order to achieve 80% power at an alpha level of 5%. Data collection continued until this target was achieved.

Data were analysed using repeated measures ANOVA, with gas (air, CO_2_) as a within-subjects factor for all analyses. For behavioural data, there was an additional within-subjects factor of ethnicity. Dependent variables (DVs) were hits (i.e., number of times participant correctly identified a previously seen face) and false alarms (i.e., number of times participant incorrectly identified a previously unseen face as seen). Additional DVs were reaction time (ms) for correct responses only, and confidence ratings (1–7) (both collapsed across seen and unseen faces). In exploratory analyses we investigated the main effects further using signal detection parameters. Data were collapsed across ethnicity and gas conditions and sensitivity (d′) and response bias (c) scores were calculated for ethnicity (own, other) and gas (CO_2_, air) separately. For each factor (i.e., ethnicity and gas), paired t-tests were conducted on sensitivity and response bias data.

In order to check the validity of the anxiety manipulation, we compared subjective (STAI-S, PANAS) and physiological (SBP, DBP, HR) responses to the CO_2_ versus air inhalations using paired t-tests. Data were checked for normality prior to analyses. A random selection of questionnaire data (20% of total) that were entered into SPSS were checked for accuracy by an independent rater. There was good reliability, with an error rate of 0.01%.

## 3 Results

### 3.1 Characteristics of participants

Participants (*n* = 30; 50% male) were aged between 18 and 32 years (*M* = 22, *SD* = 3) and had BMIs between 19 and 29 (*M* = 23, *SD* = 3). STAI-trait anxiety scores and ASI scores ranged between 25 and 54 (*M* = 34, *SD* = 7) and 1 and 37 (*M* = 14, *SD* = 7) respectively. EPQ-R scores ranged between 1 and 16 (*M* = 7, *SD* = 3) for psychoticism, 1 and 21 (*M* = 9, *SD* = 5) for neuroticism, and 5 and 21 (*M* = 14, *SD* = 4) for extraversion.

### 3.2 Face memory

#### 3.2.1 Identification accuracy (hits)

**Fig. 1:**
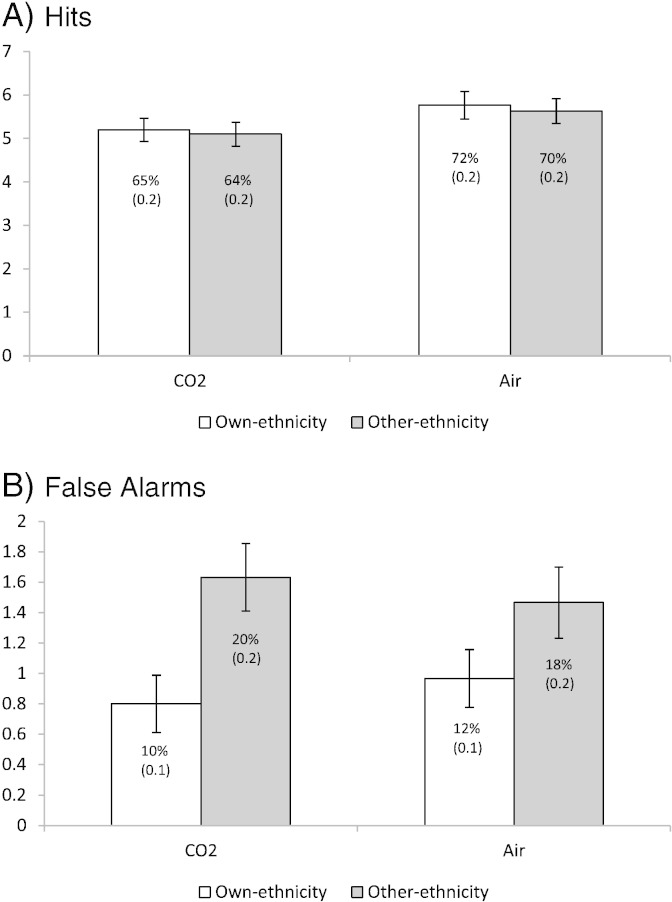
Mean (error bars show standard error) hits (panel A) and false alarms (panel B) for own-ethnicity and other-ethnicity faces during CO_2_ and air inhalations. To aid comparison with other studies, percentages are provided in chart with standard deviations given in parenthesis.

There was a main effect of gas for accuracy of identifying faces seen previously (*F*[1,29] = 5.52, *p* = .026, *η*_*p*_^2^ = .16) with lower accuracy in the CO_2_ condition (*M* = 5.2, *SD* = 1.3) compared to air condition (*M* = 5.7, *SD* = 1.1). There was no evidence of a main effect of ethnicity (*p* = .71) or a gas × ethnicity interaction (*p* = .94) (see Fig. 1).

#### 3.2.2 False alarms

There was a main effect of ethnicity for false alarms (i.e., incorrectly identifying a face not seen previously) (*F*[1,29] = 11.32, *p* = .002, *η*_*p*_^2^ = .28) with more false alarms in response to other-ethnicity faces (*M* = 1.5, *SD* = 1.0) compared to own-ethnicity faces (*M* = 0.9, *SD* = 0.8). There was no evidence of a main effect of gas (*p* = 1.0) or a gas × ethnicity interaction (*p* = .35) (see Fig. 1).

#### 3.2.3 Reaction time

For correct identification of seen faces, there was a main effect of gas (*F*[1,29] = 6.28, *p* = .018, *η*_*p*_^2^ = .18), with faster responses in the air condition (*M* = 2072 ms, *SD* = 536 ms) compared to CO_2_ (*M* = 2349 ms, *SD* = 803 ms). There was no clear evidence of a main effect of ethnicity (*p* = .11) or a gas × ethnicity interaction (*p* = .11).

For correct omission of previously unseen faces, there was a main effect of ethnicity (*F*[1,29] = 4.64, *p* = .040, *η*_*p*_^2^ = .14) with faster responses to own-ethnicity faces (*M* = 2067, *SD* = 582) compared to other ethnicity faces (*M* = 2277, *SD* = 671). There was no evidence of a main effect of gas (*p* = .36) or a gas × ethnicity interaction (*p* = .72).

### 3.3 Confidence ratings

There was a main effect of ethnicity for confidence ratings (*F*[1,29] = 42.37, *p* < .001, *η*_*p*_^2^ = .59) with higher confidence in responses to own-ethnicity faces (*M* = 5.6, *SD* = 0.6) compared to other-ethnicity faces (*M* = 5.2, *SD* = 0.5). There was no evidence of a main effect of gas (*p* = .77) or a gas × ethnicity interaction (*p* = .61).

### 3.4 Sensitivity and response bias

Sensitivity was higher for own-ethnicity faces (*M* = 1.9, *SD* = 0.7) compared to other-ethnicity faces (*M* = 1.5, *SD* = 0.6) (*t*[29] = 2.93, *p* = .006). In addition, response bias was higher for own-ethnicity faces (*p* = .10) and in the CO_2_ condition compared to air (*p* = .11), and sensitivity was lower in the CO_2_ condition compared to air (*p* = .11), but there was no clear statistical evidence for these effects.

### 3.5 Manipulation check

Compared to air, CO_2_ inhalation induced increases in heart rate (*t*[29] = 5.52, *p* < .001), SBP (*t*[29] = 3.93, *p* < .001), negative affect (*t*[29] = 4.67, *p* < .001) and state ratings of anxiety (*t*[29] = 5.83, *p* < .001), and lower positive affect (*t*[29] = 3.36, *p* = .002). There was no clear evidence of a difference in DBP between CO_2_ and air inhalations (*p* = .33) Descriptive statistics are presented in Table 1.

## 4 Discussion

This study is the first to use the CO_2_ model to test the effect of state anxiety on facial recognition at the point of memory testing. Consistent with previous findings and our hypothesis, anxiety was associated with reduced accuracy in facial recognition of unfamiliar faces. This effect was only observed for identification accuracy (i.e., hits). This suggests that in anxious situations individuals are less likely to accurately identify someone they have seen before, but are *not* more likely to falsely identify someone they have not seen before. It is interesting to note that ratings of confidence were not affected by the gas, indicating that individuals were not aware of any change in performance. This has implications for real world forensic situations, as these data suggest that anxious individuals will be less able to identify a perpetrator, but will not report lower confidence in their ability to do so.

The finding that anxiety affects identification accuracy, but not omission failures (i.e., false alarms) is reported elsewhere. In a study that required people to identify whether two simultaneously presented facial images were of the same individual, anxiety impaired performance reduced the number of accurate hits, but did not increase the number of false alarms [22]. A meta-analysis of 27 studies reported that stress at the time of encoding degraded identification performance for both target present (i.e., decreased hits) and target absent (i.e., increased false alarms) trials, but that the effect was larger when the target was present [17]. It is plausible that anxiety induces more caution and makes an individual less willing to positively identify an individual. However, in the current study positive identification did not lead to negative consequences for the target and there were no reductions in confidence during CO_2_ inhalation, both of which argue against heightened caution as an explanation of this finding. Deffenbacher and colleagues [17] suggest that stress at the time of encoding degrades the quality of the memory representation. In line-up situations the target is often presented alongside individuals with similar visual characteristics such as height and hair colour. Therefore, to positively identify a target, the viewer must recall detailed visual memory representations that are degraded in the stressful condition. It is therefore possible that anxiety may interfere with the recall or utilisation of the detailed memory trace that is required to maintain optimal identification accuracy. In contrast, decision of absence may rely on such detail to a lesser degree and some decisions can be made using superficial information.

This study also examined whether anxiety differentially affected recognition memory depending whether the person to-be-identified was of the same ethnicity as the viewer. We found greater false alarm rates for other-ethnicity (compared to own-ethnicity) images. We also found increased sensitivity for own-ethnicity faces using signal detection parameters. This finding is consistent with previous reports that individuals are better at identifying people of the same ethnicity as themselves and are more likely to misidentify someone of another ethnicity [23,26]. However, this effect was not altered by anxiety. There was evidence that participants were aware of their poorer performance for other-ethnicity faces, as they reported lower confidence ratings in their decisions of these faces. Due to the time restrictions associated with the inhalations, we were not able to conduct a fully balanced investigation of ethnicity. It is therefore plausible that the effects observed here are due to specific characteristics of the images/ethnicity used in the task. We have little reason to believe that this is the case, but these effects should nevertheless be replicated in a fully balanced design.

There are some limitations of this study and avenues for further research that are worthy of consideration. First, while the findings from this study have important real world implications, our task was a standard face recognition task that has subtle differences from eyewitness situations. Specifically, our participants were asked to remember multiple faces in phase one of the task and later identify if a new set of faces had been previously displayed (i.e., when novel, previously unseen faces were added). Eyewitness identification involves many of the same encoding and recognition processes, but the witness will tend to view a small number of faces initially and be asked to identify them within a multi-display line up. It is plausible that stress may have different effects on these two types of paradigm and future research should replicate these findings using an eyewitness task. Second, the stimuli used in this study were static images of male faces, and the time between encoding and recognition time phases was short (approximately 20 min). To better model real world scenarios, future studies should replicate these findings with longer between-phase intervals, and include mixed-gender line-ups. In addition, video footage could be used instead of static images, particularly during the encoding phase. The primary objective of this study was to examine the effects of acute anxiety on performance at the point of recall, but future, research could investigate the effects of anxiety on performance when it is experienced during both encoding and recall/recognition phases.

The current findings extend the literature by showing that anxiety at the time of recall leads to degraded identification accuracy even when encoding is not affected by anxiety. In addition, while these findings support the other-ethnicity effect (i.e., greater false alarms for other-ethnicity faces), we showed that acute anxiety at the point of identification did not alter this effect. This work has implications for military and forensic situations of eyewitness memory, suggesting that witness performance will be negatively affected if they are in a state of acute anxiety. The effects reported here should be replicated and expanded in future research, but these findings are the first step in this area, and introduce the 7.5% CO_2_ model of anxiety induction as a viable tool to investigate these effects. The findings suggest that anxiolytic interventions may be useful for improving eyewitness accuracy in real world situations, and this should be explored in future research.

## Author contributions

ASA and JCC developed the study concept. All authors contributed to the study design and protocol development. Testing and data collection were conducted by ASFK. ASA performed the data analysis and drafted the manuscript. All authors contributed to the interpretation of results and critical review of the manuscript. All authors approved the final version of the manuscript for submission.

## Figures and Tables

**Table 1:** Cardiovascular responses, anxiety and mood during air and CO_2_ inhalations. Standard deviations (*SD*) are given in parenthesis.

	Air	CO_2_
	Mean (*SD*)	Mean (*SD*)
Systolic BP	107.9 (9.4)	114.1 (12.2)
Diastolic BP	68.5 (6.9)	70.5 (10.3)
Heart rate	61.8 (10.1)	72.3 (12.4)
STAI state	35.1 (8.3)	48.4 (11.1)
PANAS positive	27.8 (7.4)	23.7 (7.6)
PANAS negative	11.8 (2.9)	17.1 (6.5)
